# Structural insight into the substrate recognition and transport mechanism of amino acid transporter complex ACE2-B^0^AT1 and ACE2-SIT1

**DOI:** 10.1038/s41421-023-00596-2

**Published:** 2023-09-08

**Authors:** Yaning Li, Yiming Chen, Yuanyuan Zhang, Yaping Shen, Kangtai Xu, Yaqi Liu, Zilong Wang, Renhong Yan

**Affiliations:** 1 Center for Infectious Disease Research, Westlake Laboratory of Life Sciences and Biomedicine, Key Laboratory of Structural Biology of Zhejiang Province, School of Life Sciences, Westlake University, Hangzhou, Zhejiang China; 2 Beijing Advanced Innovation Center for Structural Biology, Tsinghua-Peking Joint Center for Life Sciences, School of Life Sciences, Tsinghua University, Beijing, China; 3 School of Medicine, Southern University of Science and Technology, Shenzhen, Guangdong China; 4 Key University Laboratory of Metabolism and Health of Guangdong, Southern University of Science and Technology, Shenzhen, Guangdong China

**Keywords:** Cryoelectron microscopy, Mechanisms of disease

Dear Editor,

B^0^AT1 (SLC6A19) and SIT1 (SLC6A20) are special members of the SLC6 family that rely on the ancillary protein Angiotensin-converting enzyme 2 (ACE2) or Collectrin for their membrane trafficking^1–4^. ACE2 functions as a peptidase that regulates the maturation of angiotensin (Ang) and serves as the critical host receptor for SARS-CoV-2, while Collectrin is a homolog of ACE2 with a similar tissue distribution, including the liver and kidneys^4–6^. B^0^AT1 is responsible for the uptake of neutral amino acids such as glutamine (Gln), leucine (Leu), and methionine (Met)^7^. Mutations in the *SLC6A19* gene have been associated with a rare genetic disorder called Hartnup disease, characterized by impaired absorption of neutral amino acids in the small intestines and kidneys, leading to skin rashes, neurological symptoms, and even psychiatric disorders^7–9^. Studies on *SLC6A19* knockout mice have shown decreased availability of neutral amino acid and improved glycaemic control, suggesting B^0^AT1 as a potential drug target for type 2 diabetes^10^.

On the other hand, SIT1 primarily facilitates the transport of imino acids such as proline (Pro) and pipecolate^11^. Mutations in the *SLC6A20* gene have been linked to a rare genetic disorder known as iminoglycinuria. This disorder is characterized by impaired renal reabsorption of imino acids, resulting in excessive excretion of these imino acids into the urine^12^. Furthermore, the expression levels of *SLC6A20* are closely associated with the risk and severity of COVID-19 infection^13^. Overexpression of SIT1 has been shown to notably decrease the receptor binding domain (RBD) of SARS-CoV-2 binding to human cells, possibly by trapping ACE2 in the cytosol. This suggests a close relationship among ACE2, SIT1, and SARS-CoV-2^14^.

Despite extensive structural research conducted on the bacterial homologs of the SLC6 family, the *Drosophila* Dopamine transporter (dDAT) and the human Serotonin transporter (SERT)^15^, the molecular mechanism of B^0^AT1 and SIT1 regulated by their chaperones is less characterized. Previous studies from our research group have reported the ACE2-B^0^AT1 and ACE2-SIT1 in apo or in complex with the RBD of Spike protein in SARS-CoV-2 Omicron sub-variants, respectively, and mainly focused on the characteristics of ACE2 and RBD^5,6,14^. Here, we present the cryo-EM structures of ACE2-SIT1 bound to its native substrate Pro as well as ACE2-B^0^AT1 bound to Gln and Met, respectively.

The cryo-EM structures of the ACE2-B^0^AT1 complex bound to its native substrates Gln and Met are determined at an overall resolution of 3.2 Å and 3.1 Å, respectively. Additionally, we solved the structure of the ACE2-SIT1 complex bound to Pro at a resolution of 3.3 Å (Fig. 1; Supplementary Table S1 and Figs. S1–S4). The local resolution of the transmembrane domain of ACE2-B^0^AT1 complex in complex with Gln, Met, or ACE2-SIT1 in complex with Pro are 3.6 Å, 3.9 Å, or 3.7 Å, respectively. Despite variations in substrate specificity, these three complexes displayed striking structural similarities (Supplementary Fig. S5). In this section, we will predominantly focus on the detailed structural analysis of the ACE2-SIT1 complex bound to Pro.

**Fig. 1:**
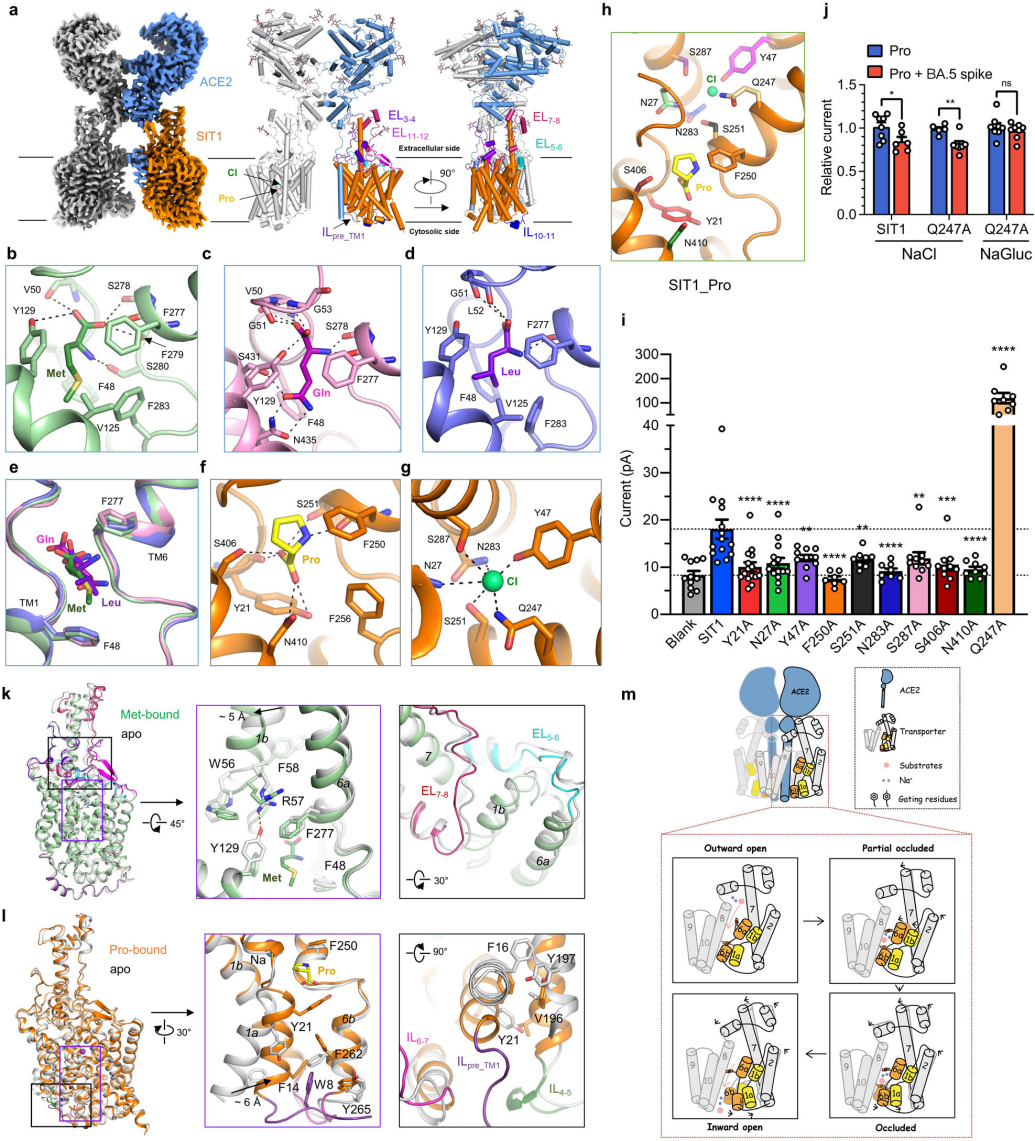
Characterization of the ACE2-B^0^AT1 and ACE2-SIT1 complex bound with natural substrates. **a** The overall cryo-EM map (left panel) and two perpendicular views (middle and right panel) of the ACE2-SIT1 complex bound with Pro. One protomer of ACE2 and SIT1 are colored blue and orange, respectively. Cl^–^ is colored green, and Pro is colored yellow. The other protomer is colored gray. The glycosylation moieties are shown as sticks. **b** The interaction interface of B^0^AT1 bound with Met. **c** The interaction interface of B^0^AT1 bound with Gln. **d** The interaction interface of B^0^AT1 bound with Leu (PDB ID: 6M17). **e** Comparison of the binding mode of the three substrates of B^0^AT1. Gln, Met and Leu are colored violet, dark-green, and deep-purple, respectively. **f** The interaction interface of SIT1 bound with Pro. **g** The interaction interface of Cl^−^ bound in SIT1. The polar interactions are shown as black dashes. **h** The binding mode of substrate Pro and Cl^−^ in SIT1. Some of the key residues that are involved in substrate binding and ion binding are shown as sticks. **i** The amplitude of 20 mM Pro induced currents in HEK-293T cells co-transfected with SIT1 or SIT1 mutants and ACE2. The difference between Y21A-N410A with WT was calculated by using one-way ANOVA and the difference between Q247A with WT was calculated by *t*-test. **j** Spike protein of BA.5 reduced Pro-induced currents on WT and Q247A and impaired when lack of chloride. The difference between treatment of Pro and co-treatment of Pro and BA.5 spike was calculated by *t*-test, respectively. Data presents mean ± SEM. **k** The structural comparison between apo (PDB ID: 6M18) and Met bound B^0^AT1 (left panel) and the detailed conformational change of B^0^AT1 once the substrate binding (middle and right panels). **l** The structural comparison between apo (PDB ID: 7Y75) and Pro-bound SIT1 (left panel) and the detailed conformational change of SIT1 during the substrate releasing process (middle and right panels). **m** The model shows a transport cycle of transporters. ACE2 is colored blue and the transporter is colored gray, orange and yellow. The red and purple balls strand for substates and Na^+^, respectively.

Our structural analysis revealed that SIT1 adopts an occluded conformation, consisting of 12 transmembrane (TM) segments arranged in a LeuT-fold (Fig. 1a). TMs 1–5 exhibit pseudo two-fold symmetry with TMs 6–10, aligned along an axis parallel to the membrane. TM1 and TM6 are interrupted in the center, forming two small helices that are connected by an uncoiled sequence, constituting the substrate-binding site. The substrate is sandwiched by two critical gating residues: Tyr21 and Phe250 (intracellular gating residue and extracellular gating residue, corresponding to Phe48 and Phe277 in B^0^AT1, respectively). The core domain is composed of TMs 1, 2, 6, and 7, while the remaining TMs form the scaffold domain. An interesting characteristic of SIT1 is the extension of the C-terminal end of TM7 into the extracellular space, where it interacts with the Collectrin-like domain (CLD) of ACE2, similar to the ACE2-B^0^AT1 complex (Supplementary Fig. S5)^5^. Additionally, SIT1 exhibits a short helix between TM10 and TM11 on the intracellular side, named IL_10-11_, and three long insertion loops, named EL_3-4_, EL_7-8_, and EL_11-12_, between TM3 and TM4, TM7 and TM8, and TM11 and TM12, respectively (Fig. 1a; Supplementary Fig. S1c, d). In comparison, B^0^AT1 has an extended intracellular loop preceding TM1 (IL_pre_TM1_) on the intracellular side, which differs from SIT1 (Supplementary Fig. S6). Notably, the interface between ACE2 and B^0^AT1 or SIT1 remains stable whether when the substrate is bound or not (Supplementary Fig. S6).

The binding pattern of amino acid substrates differs among the ACE2-B^0^AT1+Gln, ACE2-B^0^AT1+Met, ACE2-B^0^AT1+Leu, and ACE2-SIT1+Pro complexes, despite all binding to the unwound region of TM1 and TM6 (Fig. 1b–g). In the B^0^AT1 complex, Met binds in a classical mode, establishing five hydrogen bonds with the carbonyl oxygen atoms of Val50 in TM1, carbonyl oxygen atoms of Phe277 and Phe279 in TM6, the side chain of Ser278, and the side chain of Tyr129 in TM3. Additionally, the α-amino group of Met forms a hydrogen bond with the side chain of Ser280. The hydrophobic side chain of the Met substrate is surrounded by a cluster of hydrophobic residues, including Phe48, Val125 and Phe283 (Fig. 1b). On the other hand, Gln substrate is stabilized by the side chains of Ser431 and Asn435 in TM10 via hydrogen bonds, and its α-carboxylate and α-amino groups are positioned closer to the unwound region of TM1 and TM6 compared to Met (Fig. 1c). The binding mode of Leu substrate is more similar to that of Met (Fig. 1d). Neutral amino acids containing hydrophobic side chains and charged side chains might adopt different binding preference in B^0^AT1 (Fig. 1e). In contrast, the Pro-binding pocket is smaller than that of Met and Gln, with its α-carboxylate group binding to the carbonyl oxygen atoms of Tyr21, and the side chains of Ser251, Ser406, and Asn410, while its α-amino group binds to the carbonyl oxygen atoms of Phe250 (Fig. 1f). Additionally, in our structural analysis, we have identified a putative ion located on the extracellular side of SIT1, coordinated by the side chains of Asn27, Tyr47, Gln247, Ser251, Asn283, and Ser287 (Fig. 1g). Differentiating between a sodium ion and a chloride ion in this specific position is challenging based on the current structure alone. However, considering that the coordinating residues (Tyr47, Gln247, Ser251, and Ser287) are conserved in other well-known members of the SLC6 family, such as SERT, which has been shown to bind chloride ions, we have placed a chloride ion at this position (Supplementary Fig. S7).

The transport path of SIT1 is clearly characterized by the binding of Pro and a chloride ion (Fig. 1h). To examine the transport activity, mutations were introduced in SIT1 by replacing each of these residues with Ala. The transport activity was assessed by measuring the currents associated with sodium-Pro cotransport when ACE2 and SIT1 were co-expressed in HEK293T cells. All mutants, except Q247A, resulting in a marked decrease in Pro-induced currents compared with the wild-type (WT) complex (Fig. 1i; Supplementary Fig. S8). Immunostaining and western-blotting assay confirmed that the expression levels of SIT1 and the mutants were consistent (Supplementary Fig. S9). Interestingly, the Q247A mutant, which is conserved in all the SLC6 members, exhibited a dramatically increased current under the same conditions (Fig. 1i). We also conduct a dose-amplitude relationship of Pro on WT or Q247A of SIT1 and found that EC_50_ are about 2.51 mM and 0.07 mM Pro for WT and Q247A mutant, respectively (Supplementary Fig. S10). Since the function of SIT1 is closely associated with chloride, we conducted further experiments to investigate its properties. Specifically, we tested the response of both the WT and Q247A mutant using a bath solution primarily composed of NaGluc instead of NaCl. Remarkably, when chloride was absent, we observed a significant decrease of ~40% in the pro-induced current for both the WT and Q247A mutant, compared to the current recorded under normal chloride conditions. This finding suggests that the Q247A mutant remains dependent on chloride for its functionality (Supplementary Fig. S11). Additionally, considering the role of ACE2 in SARS-CoV-2 infection, we also tested whether the Spike protein of SARS-CoV-2 binding could affect the transport activity of the ACE2-SIT1 complex. Cells expressing SIT1 and ACE2 were treated with Pro at a dose that can partly activate the complex at first and then treated with the same dose of Pro with the addition of Spike protein of SARS-CoV-2 (Supplementary Fig. S12). We also conducted experiments involving the trimeric Spike protein from Omicron subvariant BA.5. We incubated both the WT and Q247A mutant with the BA.5 Spike protein and observed a reduction of ~20% in current for both variants (Fig. 1j; Supplementary Fig. S12). This suggests a potential role of SARS-CoV-2 infection in influencing the transport activity of the ACE2-SIT1 complex. Interestingly, when we removed chloride, the BA.5 Spike protein did not exhibit any inhibitory effect on the Q247A mutant (Fig. 1j; Supplementary Fig. S12). This finding suggests that the Q247A mutant may not be affected by the Spike protein in the absence of chloride.

Additionally, we introduced two triple mutants into the SIT1 protein, namely N319A&D322A&D325A (referred to as NDD) and W111A&H115A&W168A (referred to as WHW), to investigate their impact on the interaction between ACE2 and SIT1. The results demonstrated that disrupting the interaction between SIT1 and ACE2 led to a significant decrease in Pro-induced currents (Supplementary Fig. S13). This highlights the critical importance of the interaction between ACE2 and SIT1 for the proper functioning of the complex.

The substrate binding and release process plays a crucial role in the completion of the transport cycle. The different states observed in the SLC6 family members provide valuable insights into the transport mechanism (Fig. 1k; Supplementary Fig. S14). Upon substrate binding, dramatic conformational changes occur in the ACE2-B^0^AT1+Met complex compared to the apo ACE2-B^0^AT1 complex (Fig. 1k). Specifically, the hydrogen bonds between Arg57 and Tyr129 in apo ACE2-B^0^AT1 complex are disrupted once the substrate Met binding and the guanidinium group of Arg57 is oriented towards the benzene group of Phe277, forming a cation-π interaction, which triggers the rotation of TM1b by ~5 Å towards the extracellular end and thus causes the shift of the C-terminal end of the EL_7-8_ and EL_5-6_ (Fig. 1k). It is important to note that the order of movement in TM1b and TM6 described here may be reversed in the actual working state.

The inward-open conformation of ACE2-SIT1 bound with BA.5 RBD and the occluded conformation of ACE2-SIT1+Pro provide important clues for substrate release (Fig. 1l). In the occluded conformation, Trp8 of IL_pre_TM1_, Phe14 of TM1a, Phe262 and Tyr265 in TM6b enclose a π-π interaction network (Fig. 1l). During the substrate release process, the side chain of the intracellular gating residue Tyr21 in TM1a undergoes a large movement, which triggers the rotation of Tyr265 in TM6b, opening the intracellular vestibule. This movement disrupts the π-π interaction network, and TM1a is eventually stabilized by hydrophobic interactions involving Phe16 and Val196 and Tyr197 of TM5. Following this, IL_4-5_, IL_pre_TM1_, and IL_6-7_ undergo rearrangements (Fig. 1l).

The binding of amino acid substrates in the traditional substrate-binding site of ACE2-B^0^AT1 and ACE2-SIT1 could induce conformational changes in its neighboring region. These changes are reminiscent of the rocking motion observed in the core domain of LeuT-fold transporters, which alternate between outward-facing and inward-facing conformations during the transport cycle. Based on these observations, we tried to propose a common working model for the ACE2-B^0^AT1 complex and ACE2-SIT1 complex (Fig. 1m). In contrast to the LAT1-4F2hc complex, where the contact is restricted to the scaffold domain of LAT1, the CLD of ACE2 directly interacts with TM7 of the core domain in B^0^AT1 and SIT1. This suggests that ACE2 may play a direct role in regulating the transport activity of ACE2 on B^0^AT1 and SIT1. Upon substrate binding, the EL_7-8_ and TM1b went through noticeable movements, thus further closing the extracellular transport path. Subsequently, the intracellular gating residue Tyr21 in TM1a experiences a large movement and opens the intracellular vestibule, triggering the release of the substrate (Fig. 1m).

Our findings revealed the importance of a critical coordinating residue, Gln247 in SIT1 (corresponding to Gln274 in B^0^AT1), which is highly conserved among members of the SLC6 family. This residue is believed to play a role in regulating the sodium transport activity of SIT1. Interestingly, in both the apo state and the Pro-bound state of SIT1, the position and coordination of the ion remain similar (Supplementary Fig. S14). The analysis of chloride-deprived transport activity with the Q247A mutant strongly indicates that chloride is still necessary for the transport activity of this mutant. This suggests the vital role of chloride ions in the functionality of the ACE2-SIT1 complex. Comparisons made with the structure of the serotonin transporter (SERT) provide additional insights into the significance of Gln247 and the coordination of chloride ions. In the SERT structure, the chloride ion is observed to slightly shift into the binding site compared to SIT1, and the corresponding conserved residue, Gln332, directly interacts with a chloride ion (Supplementary Fig. S15).

However, despite the evidence supporting the likelihood of chloride as the coordinating ion, we cannot exclude the possibilities of a sodium ion being present in this position or Gln247 binding to a sodium ion in an alternative conformation. We speculated that the Gln247 might play an important role in the coordination of sodium and chloride ions, once mutated into alanine, it might lose the control of substrates and ions balance. Further investigations are necessary to fully elucidate the nature of the ion coordinating with Gln247 and its potential role in the transport mechanism of the ACE2-SIT1 complex. Intriguingly, we also found that BA.5 Spike protein of SARS-CoV-2 could reduce the Pro-stimulated current in both WT and the Q247A mutant, which suggests that SARS-CoV-2 infection might regulate the transport activity of the ACE2-SIT1 complex. Taken together, the structure determination and analysis in this work represent a step toward a detailed, mechanistic understanding of SLC6 family members and related diseases.

### Supplementary information

Supplementary Information

## Data Availability

The structures of the ACE2-SIT1 complex bound with proline (PDB: 8I91, whole map: EMD-35254, map focused on the extracellular domain: EMD-35260, map focused on transmembrane domain: EMD-35261), the ACE2-B^0^AT1 complex bound with glutamine (PDB: 8I92, whole map: EMD-35255, map focused on the extracellular domain: EMD-35262, map focused on transmembrane domain: EMD-35265) and the ACE2-B^0^AT1 complex bound with methionine (PDB: 8I93, whole map: EMD-35256, map focused on extracellular domain: EMD-35271, map focused on transmembrane domain: EMD-35273) have been deposited to the Protein Data Bank (http://www.rcsb.org) and the Electron Microscopy Data Bank (https://www.ebi.ac.uk/pdbe/emdb/), respectively. The other PDB and EMDB IDs can be found in Supplementary information, Table S1. Correspondence and requests for materials should be addressed to R.Y. (yanrh@sustech.edu.cn).
