# Supplementary material for: Structural insight into the substrate recognition and transport mechanism of amino acid transporter complex ACE2-B0AT1 and ACE2-SIT1
Source: Cell Discov. 2023 Sep 8;9:93. doi: 10.1038/s41421-023-00596-2 (PMC10491607; doi:10.1038/s41421-023-00596-2)
Supplement: Supplementary file 1 — Supplementary Information [file 41421_2023_596_MOESM1_ESM.pdf]

Methods

Protein expression and purification

The cDNAs for full-length human SIT1 (accession number: NM_020208.3), B^0AT1 (accession number: NM_001003841) and ACE2 (accession number: NM_001371415) were subcloned into pCAG respectively. An N-terminal FLAG tag was fused to SIT1 and B^0AT1, and 10xHis was fused at the C-terminal of ACE2 using a standard PCR process.

The recombinant protein was overexpressed using the HEK293F mammalian cells at 37°C under 5% CO_2 in a Multitron-Pro shaker (Infors, 130 rpm). When the cell density reached about 2.0 $\times 10^6$ cells/mL, the plasmid was transiently transfected into the cells. All the plasmids used to transfect cells were prepared by GoldHi EndoFree Plasmid Maxi Kit (CWBIO).

To express the secreted ECD of spike protein from SARS-CoV-2 WT and Omicron BA.5 subvirant, about 1.5 mg of plasmids was premixed with 3 mg of polyethylenimines (PEIs) (Polysciences) in 50 mL of fresh medium for 15 mins before adding to cell culture. Medium was collected by centrifugation at 4000×g for 15 mins after sixty hours of transfection.

To co-express ACE2-SIT1 complex or ACE2-B^0AT1complex, about 0.75 mg plasmids for SIT1 or B^0AT1 and 0.75 mg plasmids for ACE2 were premixed with 3 mg PEIs in 50 ml of fresh medium for 15 mins before adding to cell culture.

For purification of ACE2-SIT1 complex or ACE2-B^0AT1complex, the cells were collected in buffer containing 25 mM HEPES, pH 7.0, 150 mM NaCl, and three

protease inhibitors, aprotinin (1.3 μg/ml, AMRESCO), pepstatin (0.7 μg/ml, AMRESCO), and leupeptin (5 μg/ml, AMRESCO). The membrane fraction was solubilized at 4°C for 2 hours with 1% (w/v) glyco diosgenin (GDN, Anatrace) and the cell debris was removed by centrifugation at 18,700 g for 45 mins. The supernatant was loaded to anti-FLAG M2 affinity resin (Sigma). After rinsing with the wash buffer 1 containing 25 mM HEPES, pH 7.0, 150 mM NaCl, and 0.01% GDN (w/v), the protein was eluted with wash buffer 1 plus 0.2 mg/ml FLAG peptide. The eluent was further purified by Ni-NTA affinity resin (Qiagen). After eluted with the wash buffer 1 supplemented with 300 mM imidazole, the eluent was then concentrated to about 1 mL. Then the protein mixture was subjected to size-exclusion chromatography (Superose 6 Increase 10/300 GL, GE Healthcare) in the buffer containing 25 mM HEPES, pH 7.0, 150 mM NaCl and 0.01% GDN. The peak fractions were collected and concentrated for further EM analysis.

For purification of ECD protein, the collected medium is loaded to Ni-NTA affinity resin (Qiagen). After eluted with the wash buffer 2 containing 25 mM HEPES, pH 7.0, 150 mM NaCl, supplemented with 300 mM imidazole, the eluent was then concentrated to about 1 mL and subjected to size-exclusion chromatography (Superose 6 Increase 10/300 GL, GE Healthcare) in the buffer containing 25 mM HEPES, pH 7.0, 150 mM NaCl.

Cryo-EM sample preparation and data acquisition

The protein of ACE2-SIT1 complex was incubated with 5 mM proline and ACE2-

B^0AT1 complex was incubated with 5 mM L-Met or L-Gln. Protein mixtures were concentrated to 12 mg/mL and aliquots (3.3 μl) of the mixture were placed on glow-discharged holey carbon grids (Quantifoil Au R1.2/1.3), which were blotted for 3.0 s or 3.5 s and flash-frozen in liquid ethane cooled by liquid nitrogen with Vitrobot (Mark IV, Thermo Fisher Scientific). The cryo grids were transferred to a Titan Krios operating at 300 kV equipped with Gatan K3 Summit detector and GIF Quantum energy filter. Movie stacks were automatically collected using AutoEMation[1], with a slit width of 20 eV on the energy filter and a defocus range from -1.4 μm to -1.8 μm in super-resolution mode at a nominal magnification of 81,000 ×. Each stack was exposed for 2.56 s with an exposure time of 0.08 s per frame, resulting in a total of 32 frames per stack. The total dose rate was approximately 50 $e^-/Å^2$ for each stack. The stacks were motion corrected with MotionCor2[ref.2] and binned 2-fold, resulting in a pixel size of 1.087 Å/pixel. Meanwhile, dose weighting was performed[3]. The defocus values were estimated with Gctf[4].

Data processing

Particles were automatically picked using Relion 3.0.6[ref.5-8] from manually selected micrographs. For ACE2-SIT1 bound with proline, after 2D classification, good particles were selected and subject to several cycles of heterogeneous refinement with C1 symmetry in cryoSPARC. The good particles were selected and subject to homogeneous refinement with C1 symmetry in cryoSPARC. To further improve the map quality of ACE2, the particles were subject to several cycles of 3D classified and

focused refinement in Relion, resulting in a 3D reconstruction with better quality for ACE2 part. To further improve the map quality of the transmembrane domain of SIT1, the particles were C2-symmetry expanded and subject to several cycles of 3D classified and focused refinement in Relion. For ACE2-B^0AT1bound with L-Met or L-Gln, after 2D classification, good particles were selected and subject to several cycles of 3D classification with C2 symmetry in Relion. The good particles were selected and subject to refinement with C2 symmetry. To further improve the map quality of ACE2, the particles were subject to focused refinement in Relion, resulting in a 3D reconstruction with better quality for ACE2 part. To further improve the map quality of the transmembrane domain of B^0AT1, the particles were C2-symmetry expanded and subject to several cycels of 3D classified and focused refinement in Relion.

The resolution was estimated with the gold-standard Fourier shell correlation 0.143 criterion[9] with high-resolution noise substitution[10]. Refer to Materials and Methods, Supplementary Figs. S2-S4 and Supplementary Table S1 for details of data collection and processing.

Model building and structure refinement

The model building was accomplished with Phenix[11] and Coot[12]. The atomic model of the ACE2-SIT1 (PDB ID: 7Y75) and ACE2-B^0AT1(PDB ID: 6M18) were used for the initial model of ACE2-SIT1 bound with Pro and ACE2-B^0AT1 bound with L-Met/ L-Gln and fitted into focused refined maps of the transmembrane domain and ACE2 part using MDFF (molecular dynamics flexible fitting)[13]. Each residue was manually

checked with Coot with the chemical properties taken into consideration during model building. Statistics associated with data collection, 3D reconstruction and model building are summarized in Supplementary Table S1.

Whole-cell Patch-clamp Recordings

HEK-293T Cells were cultured in high-glucose (4.5 g/L) Dulbecco's Modified Eagle's Medium (DMEM) containing 10% fetal bovine serum (Gibco, USA) and streptomycin/penicillin (Thermo Fisher Scientific, USA). When the cells grew to 40% density, transfections of SLC6A20 or mutants of SLC6A20 together with ACE2 were performed by using Lipofectamine 2000 (Thermo Fisher Scientific, USA) with 2 μg DNA for each plasmid plus 0.5 μg plasmid of GFP. 8 hours after transfection, the medium with DNA-lipid complex was removed and replaced by a fresh medium. 48 hours after transfection, the cells were seeded onto poly-D-lysine-coated coverslips and maintained at 37 °C in 5% CO_2 for 4 h before the whole-cell patch-clamp recording.

Electrophysiology recording was followed with the previous report[14]. Patch pipettes (3-7 MΩ) that were fabricated from 1.5 mm capillary glass using a P-97 puller were used to record currents with an EPC-10 USB amplifier operated by PatchMaster (HEKA Elektronik, Germany). The pipette solution contained 145 mM KCl and 2 mM Mg-ATP, adjusted to pH 8.0 with KOH. The ChoCl solution contained 140 mM ChoCl, 5 mM KCl, 2 mM $MgCl_2$, 2 mM $CaCl_2$, 10 mM HEPES and 10 mM glucose, adjusted to pH 8.0 with Tris base. In the NaCl solution, ChoCl was replaced by equimolar NaCl and adjusted to pH 8.0 with NaOH. For low Cl^- medium, NaCl was replaced by equimolar

NaGluc. The recording chamber (1000 μL) was continuously superfused at 2 ml/min. Continuous current recordings were made at a holding potential of -70 mV, low-pass filtered at 300 Hz, and sampled at 20 kHz. For Pro-induced currents recording, cells were bathed in ChoCl solution initially, and then superfused with the NaCl solution for 2 min followed by 30 senconds Pro in the NaCl solution. To detect the influence of Cl^-, the first Pro-evode current was recorded in the NaCl solution, and washed with NaGluc solution for 4 min, and then Pro-evode current was recorded in the NaGluc solution. To investigate the effect of Spike protein on the activites of SIT1, the first Pro-evode current was recorded in the NaCl solution, and washed with ChoCl solution for 4 min, and then 5 μg/ml BA.5 Spike protein together with Pro-evode current was recorded in the NaCl solution.

Immunofluorescence staining

Immunostaining was performed to check the expression level of SIT1 and its mutants. The transfected cells were fixed in 4% paraformaldehyde for 30 min at room temperature. The cells were then washed 3 times with PBS and blocked with 5% donkey serum with 0.1% Triton X-100 in PBS for 1 h at room temperature and then incubated overnight at 4°C with the primary antibody: anti-FLAG antibody (mouse, 1:1000, Sino Biological, 109143-MM13). The coverslips were washed in PBS and incubated with the secondary antibody (1:400, Cy3-Donkey Anti-Mouse, JacksonImmunoResearch, 715-165-150) for 1 h at room temperature. The coverslips were then washed with PBS and mounted in Fluroshield™ with DAPI (SIGMA, USA) and observed under a

confocal laser scanning microscope LSM 980 (Zeiss, Germany). Each group has 3 coverslips and 2 pictures were taken from each cover glass. ImageJ was used for the quantification.[15]

Western-blotting

Cells transfected with WT or Q247A mutant of SIT were lysed in RIPA lysis buffer (Beyotime, China) with protease inhibitors (Roche, Germany) and phosphatase inhibitor (Roche, Germany). The homogenate was centrifuged at 12000 rpm for 20 min and discarded the sediment. The concentration of protein was measured by an enhanced BCA protein assay kit (Beyotime, China). Samples were run on SurePAGE™ (GenScipy, China) and then transferred to polyvinylidene difluoride membranes (SIGMA, USA). Blots were incubated in 5% no-fat milk for 2 hours at room temperature for blocking, then probed with anti-β-Actin (rabbit, 1:1000, Cell Signaling Technology, 4967,) and anti-FLAG (mouse, 1:1000, CWbio, CW0287M) overnight at 4°C. After wash, the membranes were incubated with HRP–conjugated anti-rabbit antibody (goat, 1:1000, Abcam, ab205718) or HRP–conjugated anti-mouse antibody (goat, 1:1000, Abcam, ab205719) for 2 hours at room temperature. Immunoblot analysis was performed with enhanced chemiluminescent immunoblot detection reagents (NCM Biotech, China) and analyzed with ImageJ.

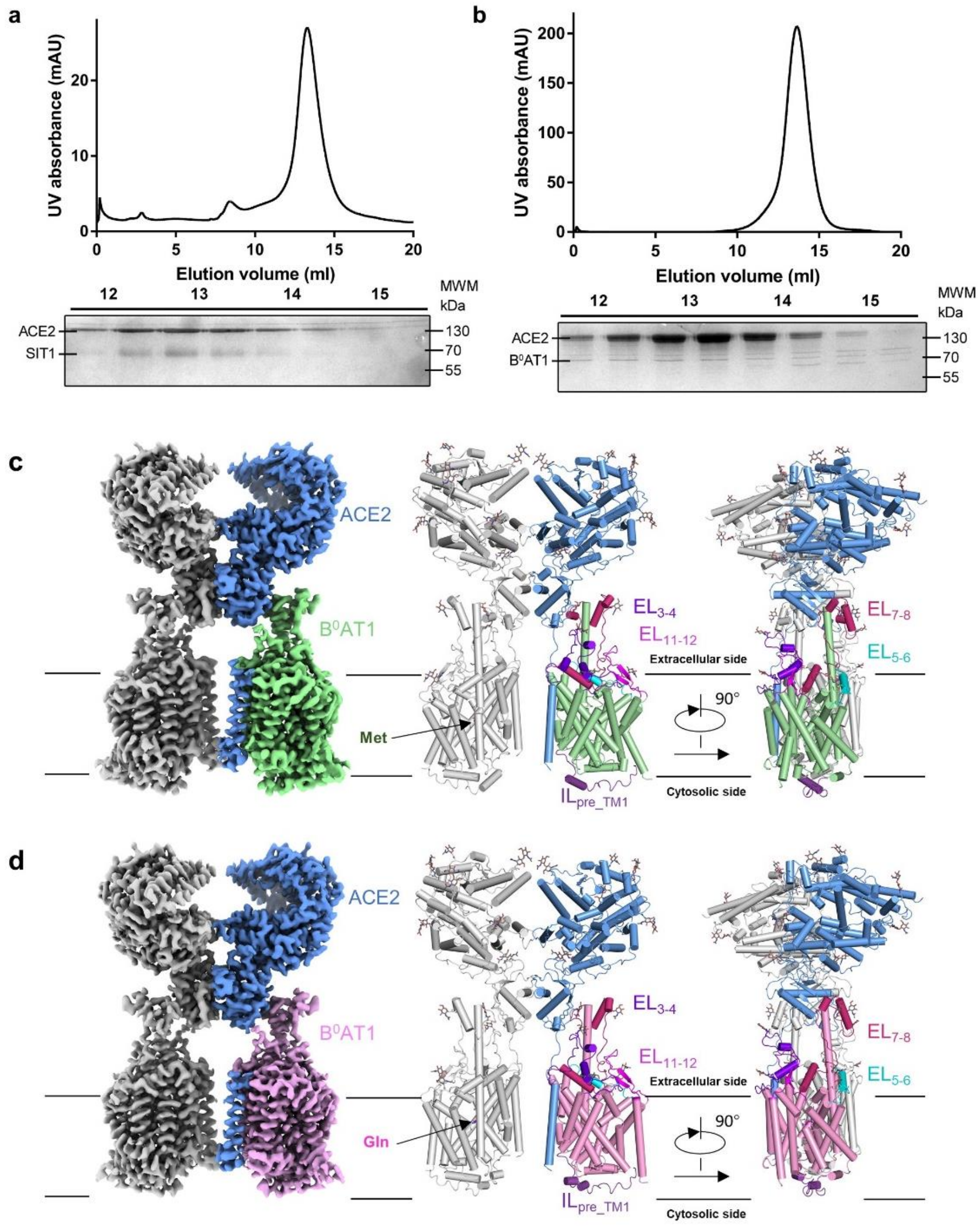

Supplementary Fig. S1 Biochemical characterization of ACE2-SIT1 and ACE2-B^0AT1.

Representative SEC purification of the ACE2-SIT1 **(a)** and ACE2-B^0AT1**(b)**. SDS–PAGE was visualized by Coomassie blue staining. **c,** The overall cryo-EM map (left panel) and two perpendicular views (middle and right panel) of the B^0AT1-ACE2 complex bound with Met. One protomer of ACE2 and B^0AT1 are colored blue and palegreen, respectively. Met is colored darkgreen. The other protomer is colored grey. **d,** The overall cryo-EM map (left panel) and two perpendicular views (middle and right panel) of the ACE2-B^0AT1complex bound with Gln. One protomer of ACE2 and B^0AT1

are colored blue and pink, respectively. Gln is colored violet. The other protomer is colored grey. The glycosylation moieties are shown as sticks.

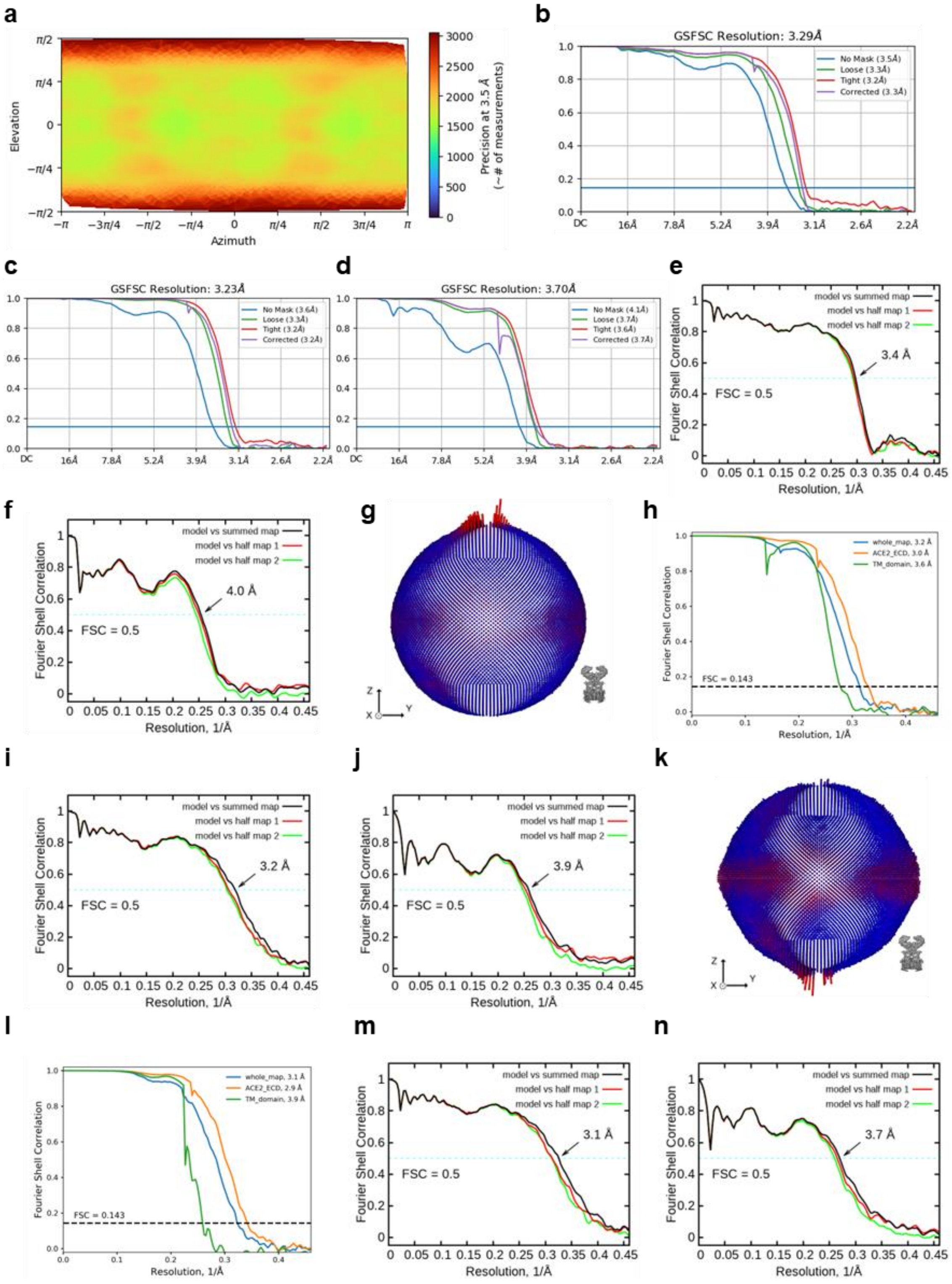

Supplementary Fig. S2 Cryo-EM analysis.

a, Euler angle distribution of the ACE2-SIT1 complex bound with Pro. **b-d,** Gold standard FSC curve of the cryoSPARC 3D reconstruction of the whole map, ECD and

TM of the ACE2-SIT1 complex bound with Pro, respectively. **e-f,** FSC curve of the refined model of the ECD and TM of ACE2-SIT1 complex bound with Pro versus the overall structure that it is refined against (black); of the model refined against the first half map versus the same map (red); and of the model refined against the first half map versus the second half map (green). The small difference between the red and green curves indicates that the refinement of the atomic coordinates did not suffer from overfitting. **g,** Euler angle distribution of the ACE2-B^0AT1 complex bound with Gln. **h,** Gold standard FSC curve of the Relion 3D reconstruction of the whole map (blue), ECD (orange) and TM (green) of ACE2-B^0AT1 complex bound with Gln, respectively. **i-j,** FSC curve of the refined model of the ECD and TM of ACE2-B^0AT1 complex bound with Gln versus the overall structure that it is refined against (black); of the model refined against the first half map versus the same map (red); and of the model refined against the first half map versus the second half map (green). The small difference between the red and green curves indicates that the refinement of the atomic coordinates did not suffer from overfitting. **k,** Euler angle distribution of the ACE2-B^0AT1 complex bound with Met. **l,** Gold standard FSC curve of the Relion 3D reconstruction of the whole map (blue), ECD (orange) and TM (green) of ACEB^0AT1 complex bound with Met, respectively. **m-n,** FSC curve of the refined model of the ECD and TM of ACE2-B^0AT1 complex bound with Met versus the overall structure that it is refined against (black); of the model refined against the first half map versus the same map (red); and of the model refined against the first half map versus the second half map (green). The small difference between the red and green curves indicates that the refinement of the atomic coordinates did not suffer from overfitting. ECD, extracellular domain. TM, transmembrane.

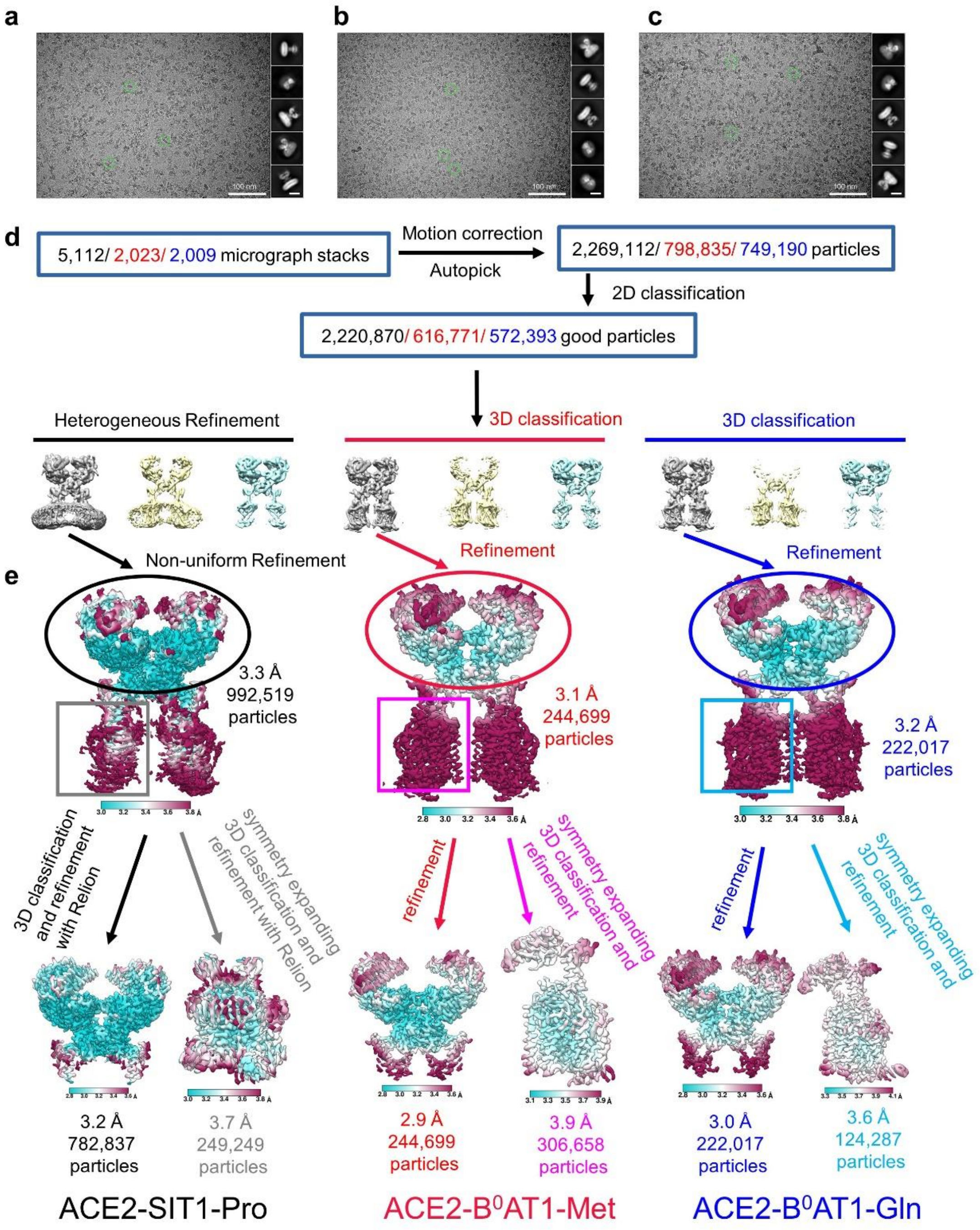

Supplementary Fig. S3 Flowchart of cryo-EM data processing.

a-c, Represent micrographs and 2D class averages of ACE2-SIT1 bound with Pro, ACE2-B^0AT1bound with Met, ACE2-B^0AT1bound with Gln. Scale bar of 2D, 10 nm. **d-e,** Flowchart for cryo-EM data processing. Please refer to the 'Data Processing' in Methods section for details. **e,** Local resolution map for the 3D reconstruction.

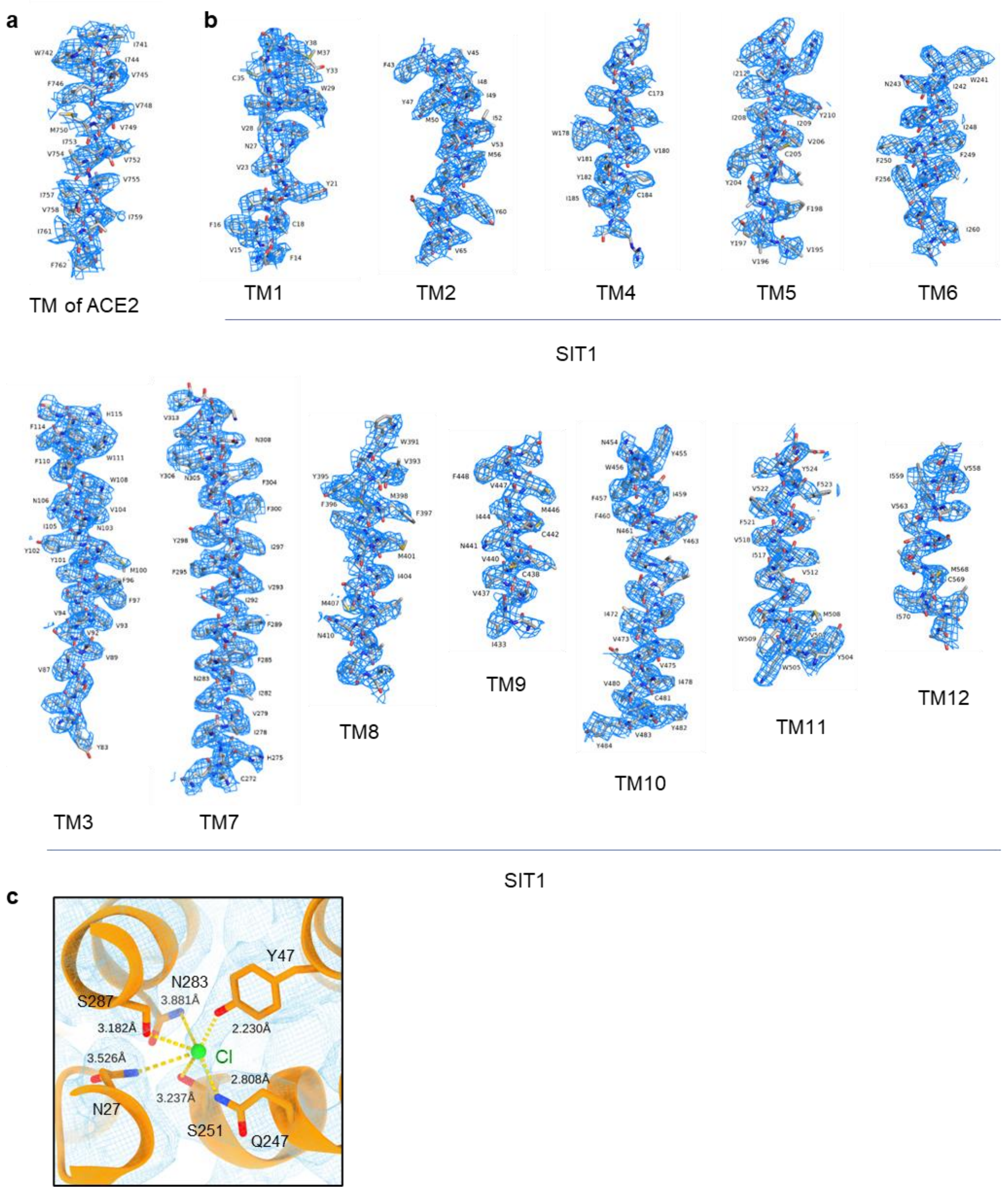

Supplementary Fig. S4 Representative cryo-EM density maps of ACE2 and SIT1.

a, Cryo-EM density map of transmembrane domain of ACE2 is shown at threshold of 7 σ. **b,** Cryo-EM density map of transmembrane domain of SIT1 is shown at threshold of 10 σ. **c,** Cryo-EM density map of Cl^- and its coordinated residues of SIT1 is shown at threshold of 7 σ.

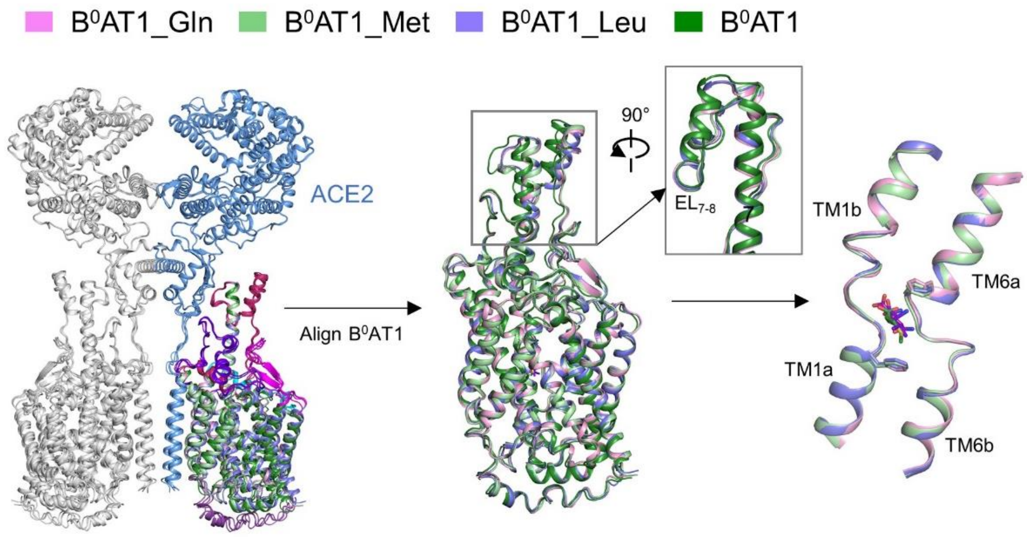

Supplementary Fig. S5 Characterization of the ACE2-B^{0}AT1complex.

Structure comparison in different B^{0}AT1, which are very similar among B^{0}AT1 bound with Gln, Met and Leu (PDB ID: 6M17). But in the extracellular region, there are a few changes between the structures bound substrate and B^{0}AT1 in the apo state (PDB ID: 6M18).

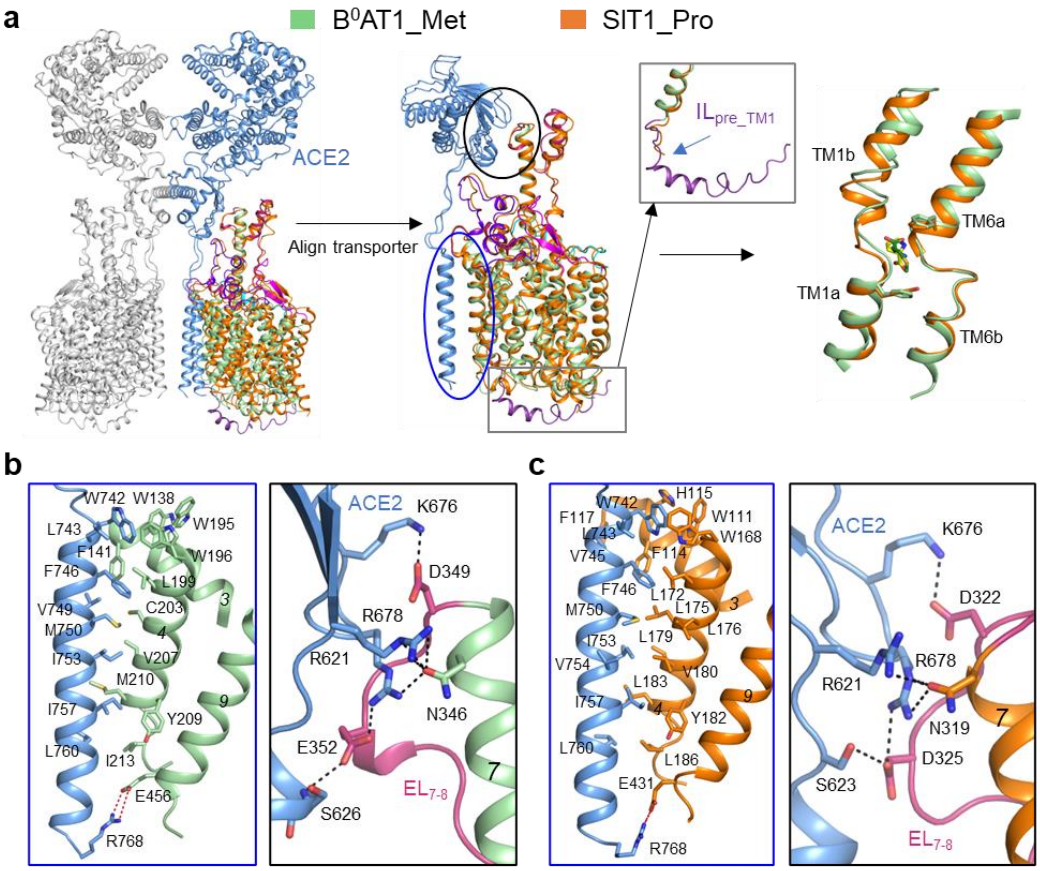

Supplementary Fig. S6 Structural comparison between ACE2-B^{0}AT1complex bound with Met and ACE2-SIT1 complex bound with Pro.

a, Structural comparison between ACE2-B^{0}AT1complex bound with Met and ACE2-SIT1 complex bound with Pro. As indicated by the blue arrow, the N-terminal of SIT1 is much shorter than B^{0}AT1, while the substrate binding mode between is similar. And the ACE2 interacts with B^{0}AT1 **(b)** and SIT1 **(c)** via two main interfaces, the extracellular interface (black circle) and the TM interfaces (blue circle). The polor interactions are shown as dashes.

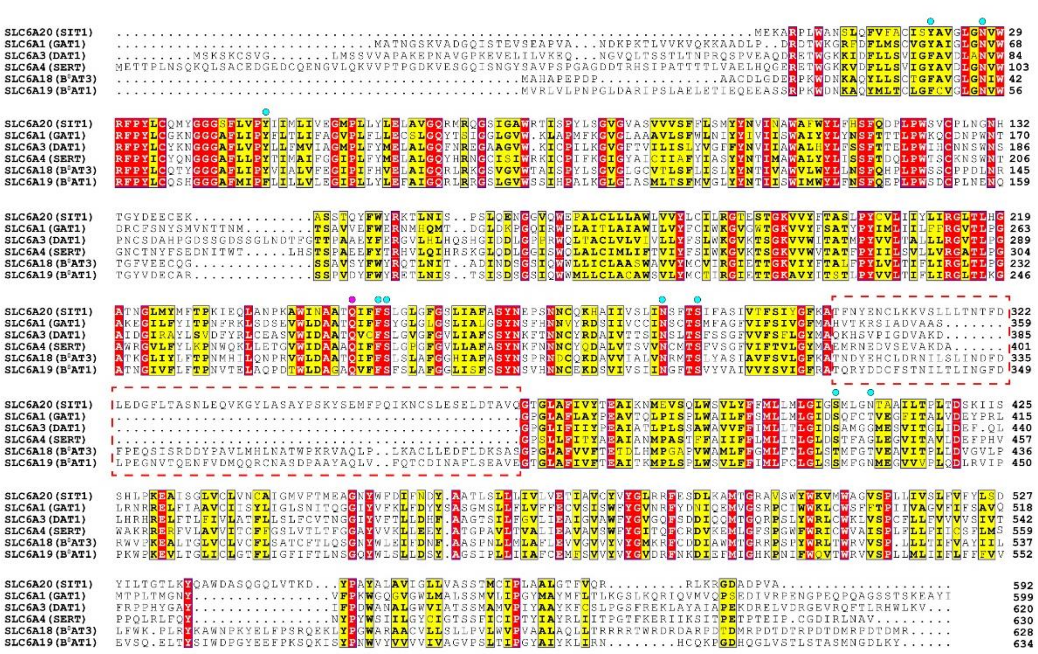

Supplementary Fig. S7 Sequence alignment of the homologs of SLC6 family from *homo sapiens*.

The six sequences are aligned using ClustalX. Identical and conserved residues are shaded red and yellow, respectively. Extented TM7 are lined out by red dotted box. Residues related to ligand binding are marked by cyan balls and Q247 are labeled by magenta ball. The Uniprot IDs for the proteins aligned are: SLC6A20: Q9NP91; SLC6A1: P30531; SLC6A3: Q01959; SLC6A4: P31645; SLC6A18: Q96N87; SLC6A19: Q695T7.

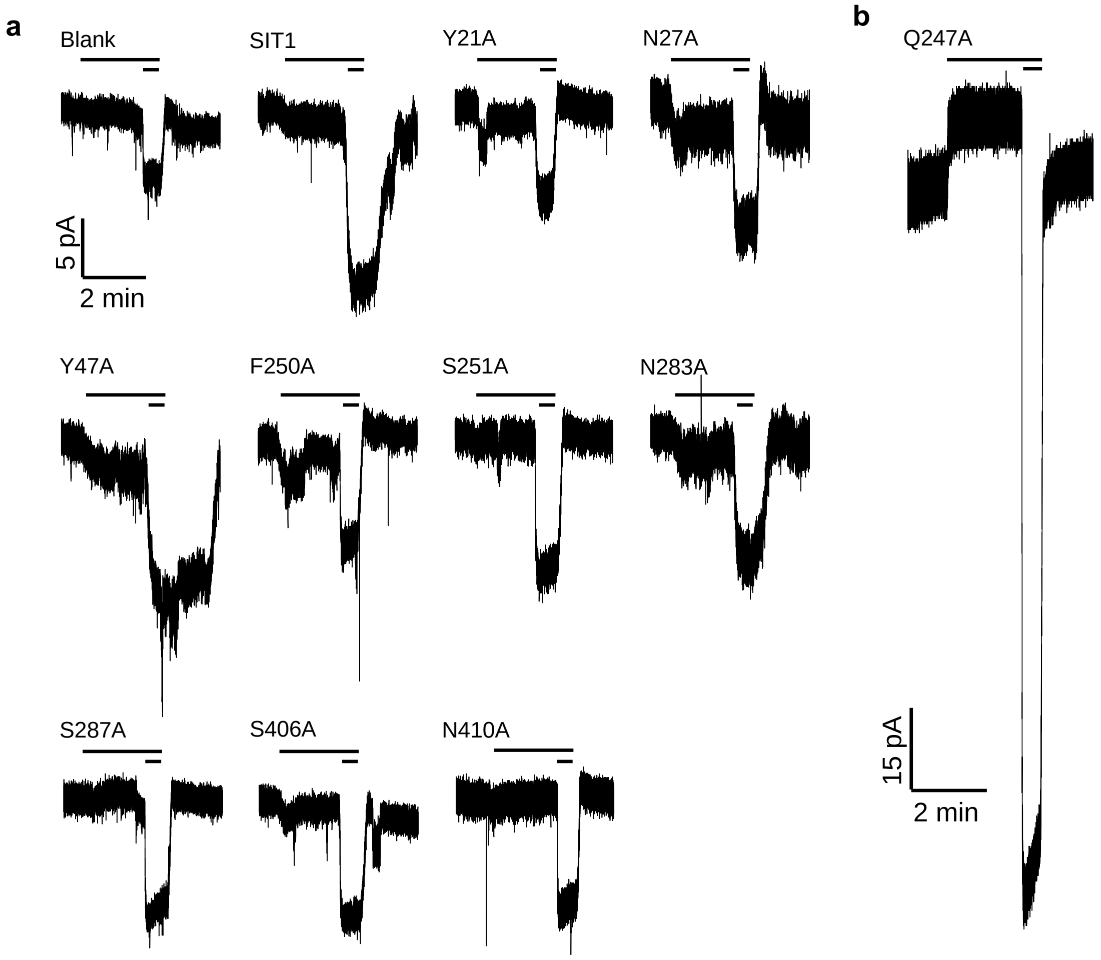

Supplementary Fig. S8 Representative traces of Pro-induced currents in HEK-293T cells co-transfected with SIT1 or SIT1 mutants and ACE2.

a, Traces from Blank HEK-293T cell, WT-SIT1 and loss-of-function mutants. **b,** Trace from gain-of-function mutant Q247A. All cells were held at -70 mV and bathed in ChoCl solution for the first three minutes. Then ChoCl solution was replaced by NaCl solution for 2.5 minutes, then 20 mM Pro was applied additionally in the last 30 seconds, and washed with ChoCl solution again.

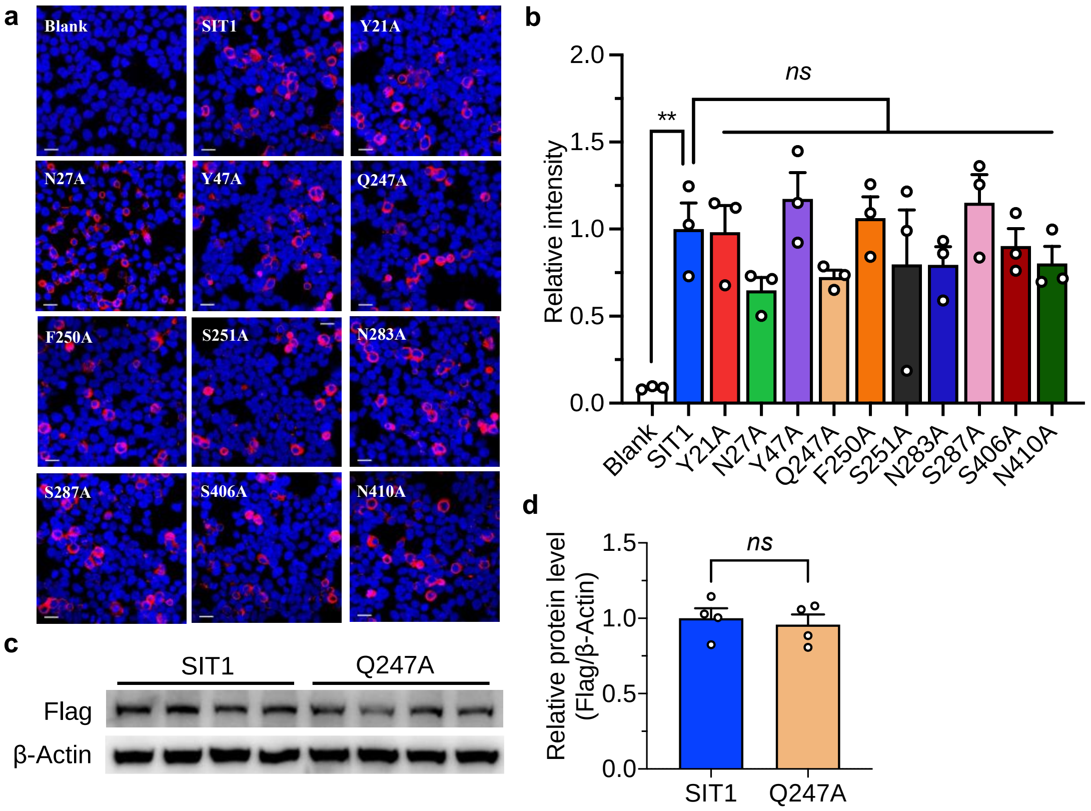

Supplementary Fig. S9 Immunostaining and western blotting of HEK-293T cells expressing SIT1 or mutants.

a, Representative images of anti-FLAG immunostaining in SIT1 and mutants transfected HEK-293T cells. Scale bar = 20 μm. **b,** Quantification of immunostaining intensity. Note the staining is absent in blank HEK293T cells and difference with WT-SIT1 was calculated by t-test.** $p < 0.01$. ns, not significant, WT vs. mutant SIT1, one-way ANOVA. Three repeated coverslips were used in each group and at least two images were collected from each coverslip for quantification. **c** and **d,** WB assays show that Q247A has a comparable expression level with WT-SIT1. ns, no significance, calculated by t-test. All data present Mean ± SEM.

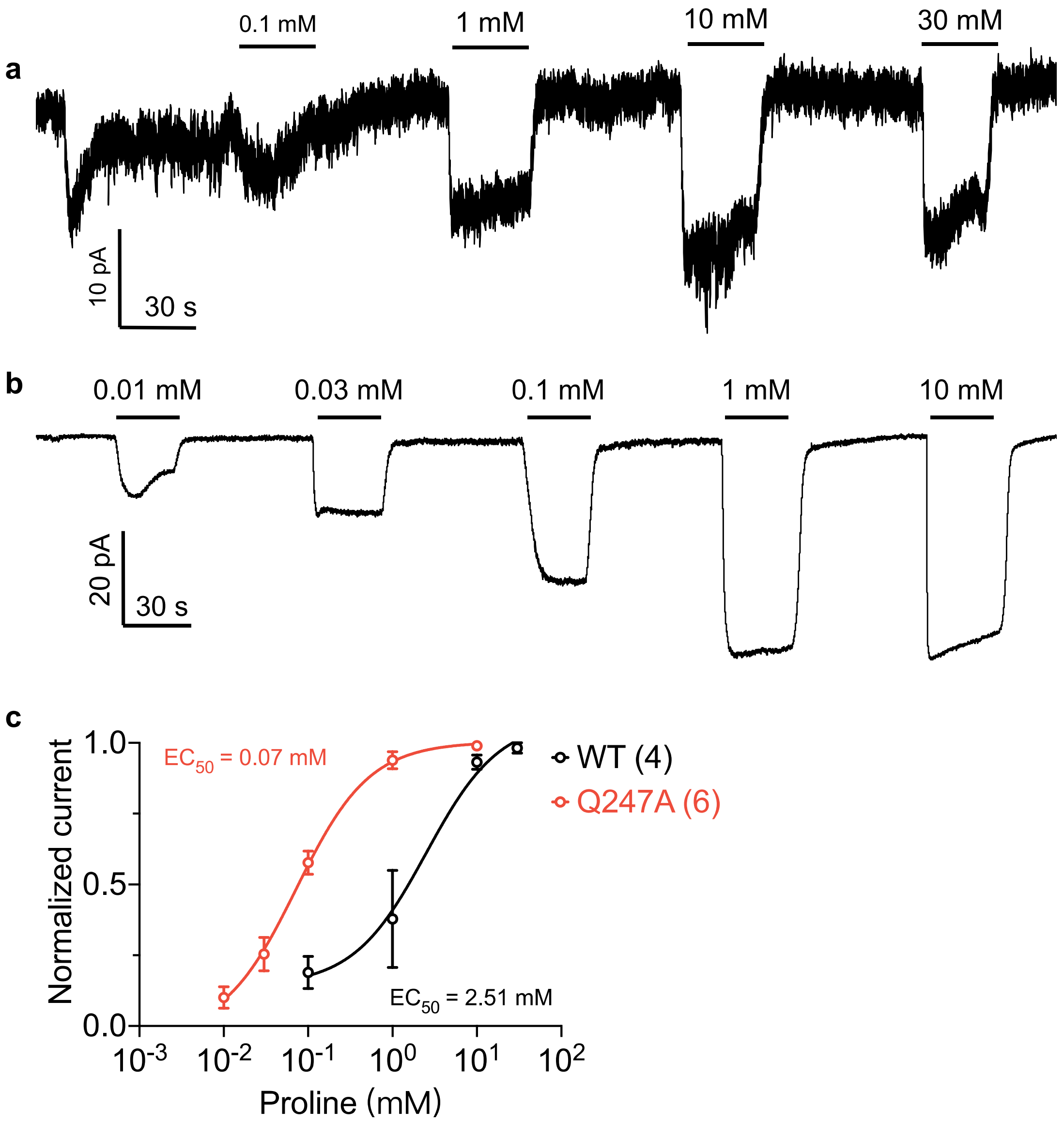

Supplementary Fig. S10 Dose-amplitude relationship of Pro on WT or Q247A of SIT1.

a, Representative trace of WT-SIT1 induced by different doses of Pro. **b,** Representative trace of Q247A induced by different doses of Pro. **c,** Dose-dependent curves of normalized currents from SIT1 and Q247A induced by Pro. Data present Mean $\pm$ SEM.

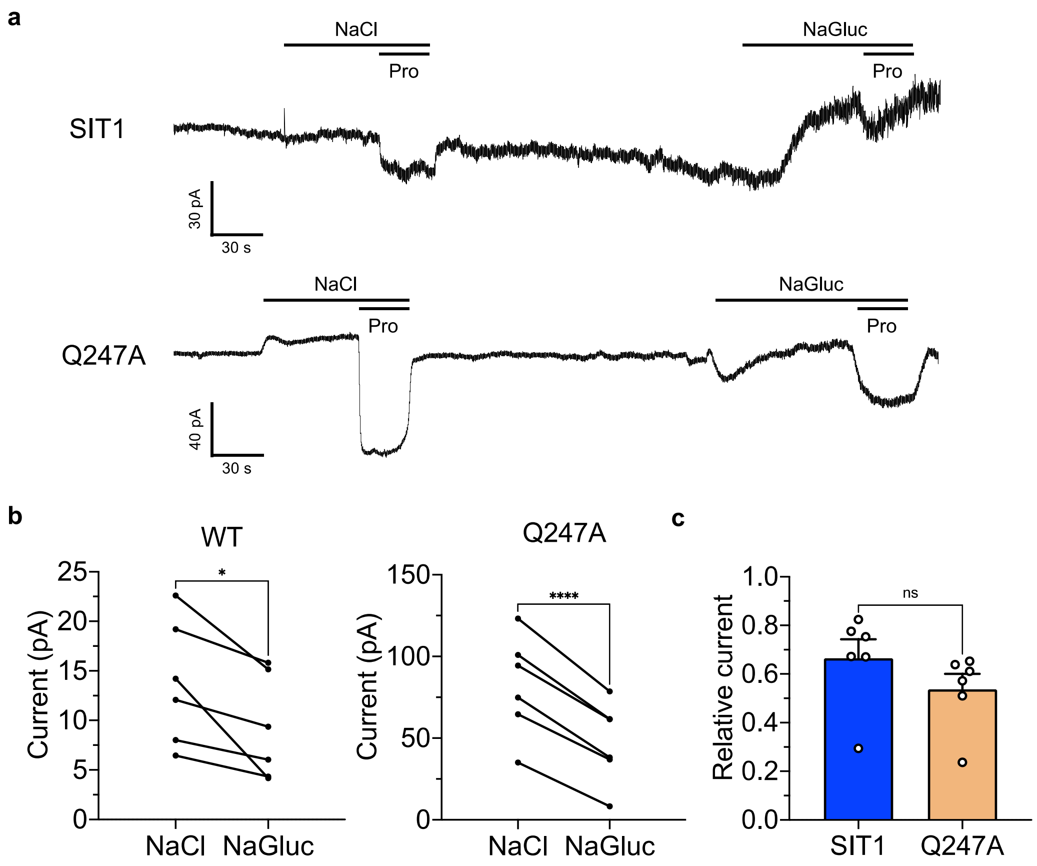

Supplementary Fig. S11 Impaired Pro-induced current with the absence of chloride.

a-b, Representative traces and summarize the result of Pro-induced current at the presence and absence of chloride. Cells were bathed in ChoCl solution initially. The difference was calculated by paired t-test. *$p < 0.05$, ****$p < 0.0001$. **c,** Relative currents induced by Pro in NaCl or NaGluc. ns, no significance, calculated by t-test. All data present Mean ± SEM.

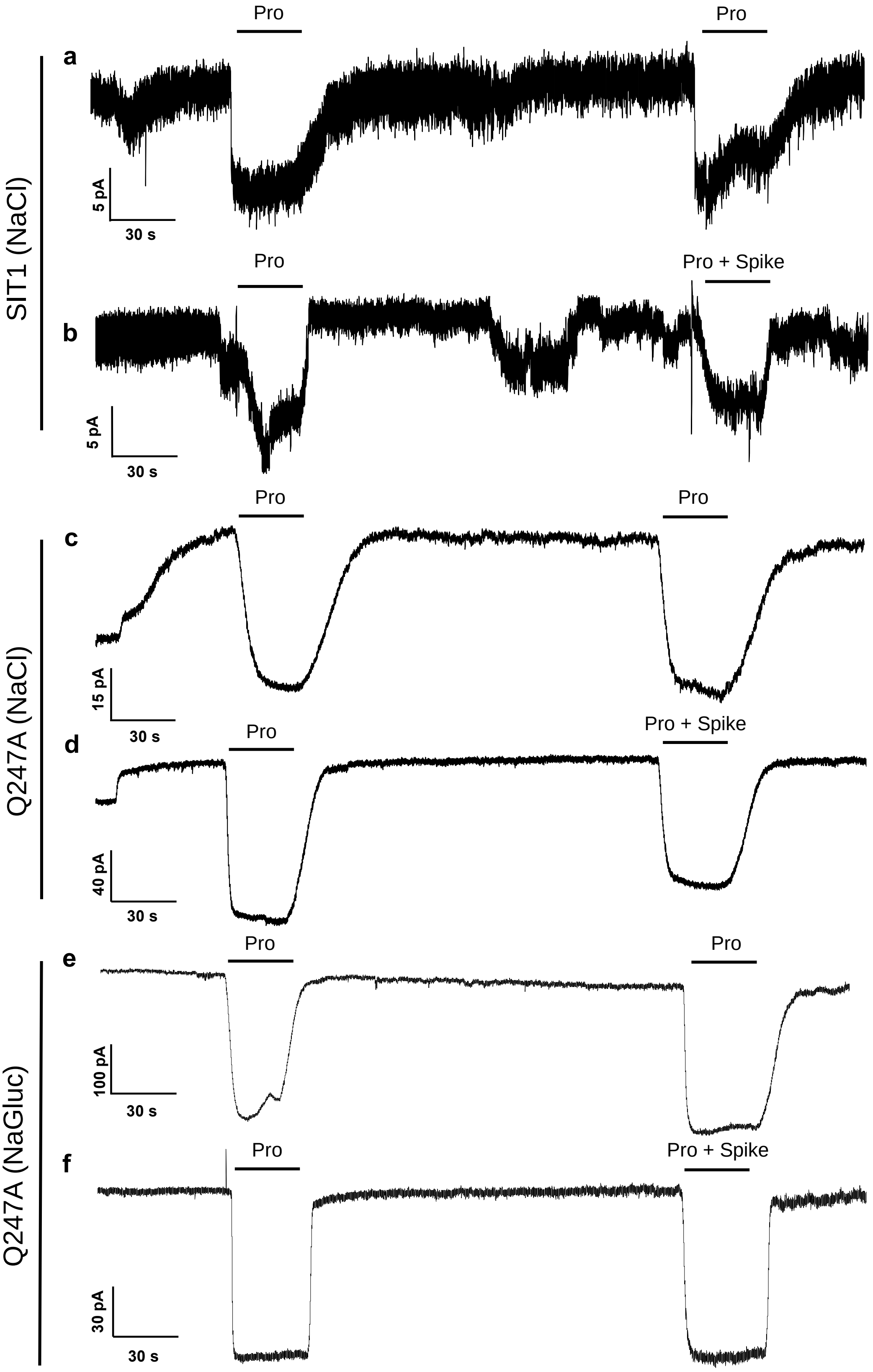

Supplementary Fig. S12 BA.5 Spike protein inhibit Pro induce current on WT-SIT and Q247A mutant.

a-b, Cells were treated with 10 mM Pro at first and then co-treated with 10 mM Pro and 5 μg/mL Spike protein of BA.5 virus. **c-d,** Cells were treated with 0.1 mM Pro at first and then co-treated with 0.1 mM Pro and 5 μg/mL Spike protein of BA.5 virus. **e-f,** Cells were treated with 0.1 mM Pro at first and then co-treated with 0.1 mM Pro and 5 μg/mL Spike protein of BA.5 virus in the NaGluc buffer.

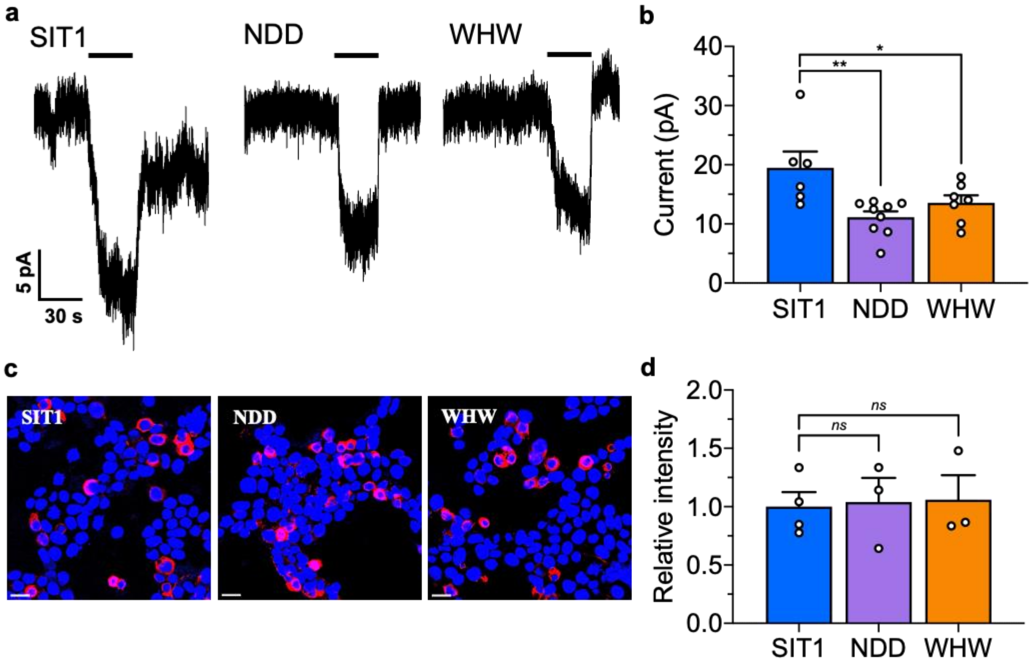

Supplementary Fig. S13 Disruption of interaction between SIT1 and ACE2 down-regulates the function of SIT1.

a-b, Traces and currents from WT-SIT1 and loss-of-function mutants. Cells were bathed in NaCl solution and treated with 20 mM Pro (show as stick). **c-d,** Anti-FLAG immunostaining of WT-SIT1 and mutants. Scale bar = 20 µm.. All Data present Mean ± SEM. ns, no significance, *$p < 0.05$, **$p < 0.01$, by One-Way ANOVA.

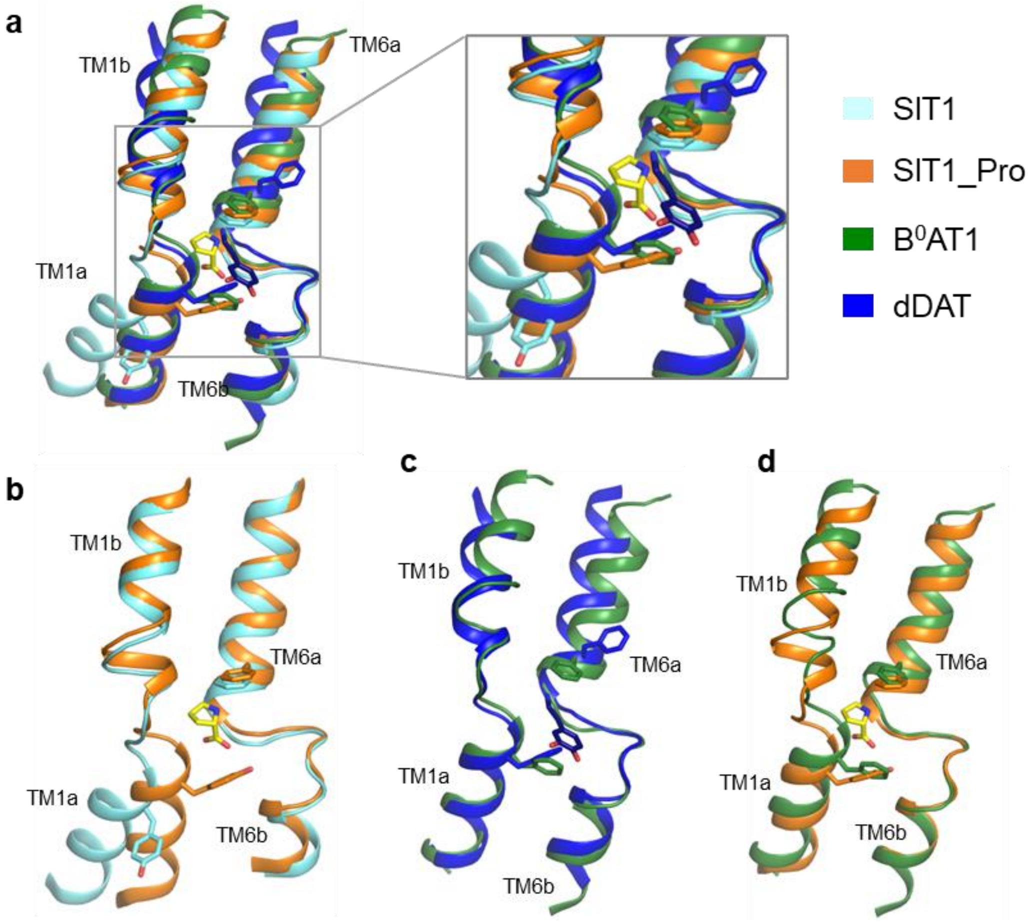

Supplementary Fig. S14 Conformational change among various states.

a, Comparison of the inward-open state of SIT1_apo (PDB ID: 7Y75), two occluded structure of SIT1 bound with Pro and B^0AT1_apo (PDB ID: 6M18), and the outward-open state of dDAT bound with dopamine (PDB ID: 4XP1). **b,** Inward-open and occluded comparison. **c,** Outward-open and occluded comparison. **d,** Two occluded conformation between B^0AT1 and SIT1 comparison.

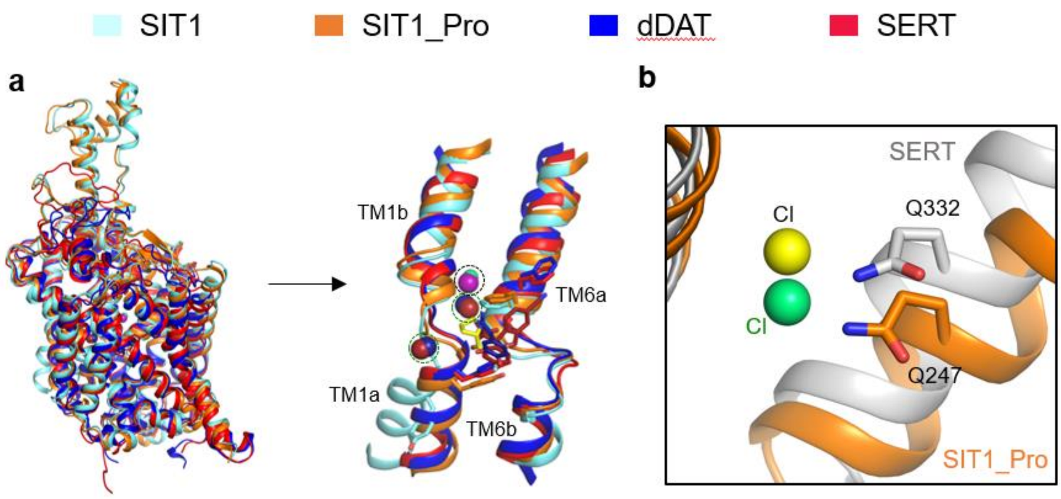

Supplementary Fig. S15 Comparison between various states in the presence of Cl^-.

a, Structural comparison among the inward-open structure of SIT1 (PDB ID: 7Y75), occluded structure of SIT1 bound with Pro, and the outward-open structure of dDAT bound with dopamine (PDB ID: 4XP1) or SERT bound with s-citalopram (PDB ID: 5I71). **b,** Structural comparison between SIT1 bound with Pro and SERT (PDB ID: 5I6X).

Supplementary Table S1 Cryo-EM data collection, refinement and validation statistics

**Data collection**			
EM equipment		Titan Krios (Thermo Fisher Scientific)	
Voltage (kV)		300	
Detector		Gatan K3 Summit	
Energy filter		Gatan GIF Quantum, 20 eV slit	
Pixel size (Å)		1.087	
Electron dose (e-/Å2)		50	
Defocus range (µm)		-1.4 ~ -1.8	
Sample	ACE2-SIT1+Pro	ACE2-B^0AT1+Gln	ACE2-B^0AT1+Met
Number of collected micrographs	5,112	2,023	2,009
**3D Reconstruction**			
Software	CryoSPARC v4	Relion 3.0.6	Relion 3.0.6
Number of used particles (Overall)	992,519	222,017	244,699
Resolution (Å) (Overall)	3.3	3.2	3.1
Symmetry	C1	C2	C2
Map sharpening B-factor (Å2)		-90	
**Refinement**			
Software		Phenix	
Cell dimensions			
a=b=c (Å)		313.056	
α=β=γ (˚)		90	
Model composition			
Protein residues	2,646	2,708	2,710
Side chains assigned	2,646	2,708	2,710
Sugar	32	38	38
Substrate	2	2	2
Zn	2	2	2
Cl	2	0	0
R.m.s deviations			
Bonds length (Å)	0.010	0.013	0.008
Bonds Angle (˚)	1.016	0.922	0.983
Ramachandran plot statistics (%)			
Preferred	92.56	92	93.04
Allowed	7.13	7.93	6.89
Outlier	0.3	0.07	0.07

Supplementary reference:

1 Lei, J. & Frank, J. Automated acquisition of cryo-electron micrographs for single particle reconstruction on an FEI Tecnai electron microscope. *Journal of Structural Biology* **150**, 69-80 (2005). https://doi.org:https://doi.org/10.1016/j.jsb.2005.01.002

2 Zheng, S. Q. *et al.* MotionCor2: anisotropic correction of beam-induced motion for improved cryo-electron microscopy. *Nature Methods* **14**, 331-332 (2017). https://doi.org:10.1038/nmeth.4193

3 Grant, T. & Grigorieff, N. Measuring the optimal exposure for single particle cryo-EM using a 2.6 Å reconstruction of rotavirus VP6. *eLife* **4**, e06980 (2015). https://doi.org:10.7554/eLife.06980

4 Zhang, K. Gctf: Real-time CTF determination and correction. *Journal of Structural Biology* **193**, 1-12 (2016). https://doi.org:https://doi.org/10.1016/j.jsb.2015.11.003

5 Zivanov, J. *et al.* New tools for automated high-resolution cryo-EM structure determination in RELION-3. *eLife* **7**, e42166 (2018). https://doi.org:10.7554/eLife.42166

6 Kimanius, D., Forsberg, B. O., Scheres, S. H. W. & Lindahl, E. Accelerated cryo-EM structure determination with parallelisation using GPUs in RELION-2. *eLife* **5**, e18722 (2016). https://doi.org:10.7554/eLife.18722

7 Scheres, S. H. W. RELION: Implementation of a Bayesian approach to cryo-EM structure determination. *Journal of Structural Biology* **180**, 519-530 (2012). https://doi.org:https://doi.org/10.1016/j.jsb.2012.09.006

8 Scheres, S. H. W. A Bayesian View on Cryo-EM Structure Determination. *Journal of Molecular Biology* **415**, 406-418 (2012). https://doi.org:https://doi.org/10.1016/j.jmb.2011.11.010

9 Rosenthal, P. B. & Henderson, R. Optimal Determination of Particle Orientation, Absolute Hand, and Contrast Loss in Single-particle Electron Cryomicroscopy. *Journal of Molecular Biology* **333**, 721-745 (2003). https://doi.org:https://doi.org/10.1016/j.jmb.2003.07.013

10 Chen, S. *et al.* High-resolution noise substitution to measure overfitting and validate resolution in 3D structure determination by single particle electron cryomicroscopy. *Ultramicroscopy* **135**, 24-35 (2013). https://doi.org:https://doi.org/10.1016/j.ultramic.2013.06.004

11 Adams, P. D. *et al.* PHENIX: a comprehensive Python-based system for macromolecular

structure solution. *Acta crystallographica. Section D, Biological crystallography* **66**, 213-221 (2010). https://doi.org:10.1107/s0907444909052925

12 Emsley, P., Lohkamp, B., Scott, W. G. & Cowtan, K. Features and development of Coot. *Acta crystallographica. Section D, Biological crystallography* **66**, 486-501 (2010). https://doi.org:10.1107/s0907444910007493

13 Trabuco, L. G., Villa, E., Mitra, K., Frank, J. & Schulten, K. Flexible Fitting of Atomic Structures into Electron Microscopy Maps Using Molecular Dynamics. *Structure* **16**, 673-683 (2008). https://doi.org:https://doi.org/10.1016/j.str.2008.03.005

14 Takanaga, H., Mackenzie, B., Suzuki, Y. & Hediger, M. A. Identification of mammalian proline transporter SIT1 (SLC6A20) with characteristics of classical system imino. *J Biol Chem* **280**, 8974-8984 (2005).

15 Suo, Y. *et al.* Structural Insights into Electrophile Irritant Sensing by the Human TRPA1 Channel. *Neuron* **105** (2020). https://doi.org:10.1016/j.neuron.2019.11.023
